# Toward a mechanistic understanding of adsorption behavior of phenol onto a novel activated carbon composite

**DOI:** 10.1038/s41598-023-27507-5

**Published:** 2023-01-04

**Authors:** Esmaeil Allahkarami, Abolfazl Dehghan Monfared, Luis Felipe O. Silva, Guilherme Luiz Dotto

**Affiliations:** 1 Department of Petroleum Engineering, Faculty of Petroleum, Gas and Petrochemical Engineering, Persian Gulf University, Bushehr, 75169-13817 Iran; 2 Persian Gulf Star Oil Company, Bandar Abbas, Iran; 3 Department of Civil and Environmental, Universidad de la Costa, CUC, Calle 58 # 55–66, Barranquilla, Atlántico Colombia; 4 Chemical Engineering Department, Federal University of Santa Maria–UFSM, Santa Maria, RS Brazil

**Keywords:** Engineering, Chemical engineering

## Abstract

In this research, the solid–liquid adsorption systems for MSAC (PbFe_2_O_4_ spinel-activated carbon)-phenol and pristine activated carbon-phenol were scrutinized from the thermodynamics and statistical physics (sta-phy) viewpoints. Experimental results indicated that MSAC composite outperformed pristine AC for the uptake of phenol from waste streams. By increasing the process temperature, the amount of phenol adsorbed onto both adsorbents, MSAC composite and pristine AC, decreased. Thermodynamic evaluations for MSAC demonstrated the spontaneous and exothermic characteristics of the adsorption process, while positive values of ΔG for pristine AC indicated a non-spontaneous process of phenol adsorption in all temperatures. In a mechanistic investigation, statistical physics modeling was applied to explore the responsible mechanism for phenol adsorption onto the MSAC composite and pristine AC. The single-layer model with one energy was the best model to describe the experimental data for both adsorbents. The adsorption energies of phenol onto both adsorbents were relatively smaller than 20 kJ/mol, indicating physical interactions. By increasing temperature from 298 to 358 K, the value of the absorbed amount of phenol onto the MSAC composite and pristine AC at saturation (Q_sat_) decreased from 158.94 and 138.91 to 115.23 and 112.34 mg/g, respectively. Mechanistic studies confirm the significant role of metallic hydroxides in MSAC to facilitate the removal of phenol through a strong interaction with phenol molecules, as compared with pristine activated carbon.

## Introduction

Phenols are important contaminants in natural waters due to their toxicity and carcinogenicity properties^1,2^. The concentration of phenol in industrial wastes varies from 50 to 2000 mg/L^3^. Phenolic compounds, even at low concentrations, are considered health-threatening organic pollutants due to their water-soluble and acidic nature^4–6^. Therefore, the elimination of these compounds from the waste streams before their discharge to the environment is vital. In this context, some biological and physicochemical methods, such as ion exchange^6,7^, adsorption^8,9^, chemical reduction^10^, solvent extraction^11,12^, and reverse osmosis^13^, were utilized to remove the phenolic-based compounds from different solutions. However, adsorption process has gained much attention due to its ease and convenience in operation, simple design, and economic considerations^10,14,15^. In this regard, various adsorbents such as activated carbon^16,17^, minerals^18,19^, and polymers^20,21^ have been applied to investigate phenol uptake. For example, Lammini et al.^22^ synthesized cobalt oxide Co_3_O_4_ and applied it for adsorption of phenol from solution. According to their results, the highest capacity and removal percentage of phenol at pH 4 were obtained 8.10 mg/g and 98%, respectively. Dehmani et al.^23^ activated Moroccan clay with sulfuric acid and used it to uptake phenol from the aqueous solution. They indicated that clay chemical activation enhanced its adsorption capability. Mohammed et al.^24^ applied Faujasite-Type Y Zeolite for the phenol uptake from aqueous solution. They found that the equilibrium data for the uptake of phenol by the developed adsorbent were in accordance with the Langmuir adsorption isotherm.

Empirical, semi-empirical, and theoretical adsorption isotherm models can predict the experimental data obtained from these studies. Therefore, the analysis of experimental data with these models provides important information for the design of the adsorption process as an operation unit and attributes further comprehension into the mechanism of the adsorption process and economic considerations^25–27^. In general, experimental data obtained in the adsorption of phenol by various adsorbents have been discussed using Freundlich, Langmuir, Dubinin–Radushkevich, Temkin, and Liu models^28,29^. However, the interpretation of phenol adsorption properties using these models is limited due to the disability of these models to understand the complete adsorption process and the parameters affecting the process. Statistical physics modeling can be applied to explain the phenol adsorption isotherms^30^ to overcome these limitations. Indeed, the analysis of experimental data via statistical physics fundaments provides useful information about the parameters related to the steric and energetic state of the adsorption process that controls the adsorption mechanism^31^. These models assume that the phenol adsorption onto the prepared adsorbent occurs by forming a variable number of layers or a constant number (1 or 2) of layers^32^. In addition, they assume that a variable number of adsorbate molecules is linked to receptor sites of the adsorbent (unit mass)^31^. In general, statistical physics models are useful tools for obtaining new interpretations about the adsorption mechanism of adsorbate onto the developed adsorbent at the molecular level.

Recently, the activated carbon (AC) has attracted great attention for wastewater treatment purposes^33–35^ due to its large surface area, high adsorption performance, micro-porous nature, and ease of application and availability. Most of the studies in the literature have been focused on the preparation of activated carbon from different types of precursors to improve the physical properties of AC. Pal et al.^36^ synthesized four AC samples from two biomass precursors namely waste palm trunk and mangrove applying KOH as an activating agent. Their results indicated that the biomass-derived activated carbons had high surface area and large pore volume leading to improve the uptake of CO_2_. However, there are the limited studies about improving the surface properties of AC. On the other hand, activated carbon is extremely difficult to separate from the solution^37^. To overcome this limitation, the introduction of ferromagnetism in mesoporous activated carbon particles has been performed for obtaining magnetic activated carbon. Magnetic activated carbon is of great interest as offers an easy way to separate the sorbent from aqueous solution for reuse and attractive catalytic performance^38,39^. Metal ferrites (MFe_2_O_4_, M = Zn, Co, Pb, Sr, etc.) have face-centered cubic structures with M^2+^ and Fe^3+^ cations filling in the tetrahedral and octahedral coordination sites. Incorporation of the mentioned metallic cations could improve the magnetic properties of the developed composite^40,41^. Therefore, the main aim of this work is to present a mechanistic study on the lead ferrite-AC composite as a new material for wastewater decontamination purposes.

This mechanistic study applied a novel PbFe_2_O_4_ spinel—activated carbon composite (MSAC) prepared by co-precipitation to remove phenol from solutions. The adsorption system MSAC-phenol was compared with pristine activated carbon from the thermodynamic and statistical physics viewpoints. Adsorption studies investigated the influence of temperature on phenol adsorption capacity, in which five isotherm curves were constructed. From the thermodynamic perspective, the variations of Gibbs free energy, enthalpy, and entropy were evaluated. Concerning the sta-phy, some models were tested, and the steric and energetic parameters were interpreted. The present research contributes to new theoretical insights on phenol removal by MSAC composite and pristine activated carbon through statistical physics modeling.

## Experimental section

### Solid–liquid adsorption system

The adsorbents used were PbFe_2_O_4_ spinel -activated carbon composite (MSAC) prepared by co-precipitation and pristine activated carbon. Pristine AC (received from Korea; Hanil, P60,) was applied in its original form with no any purification. The adsorbate used was phenol. First, the stock solution of phenol (Merck, Darmstadt, Germany) was prepared by dissolving specific amounts of adsorbate into double-distilled water. Then, the desired concentrations of phenol for doing adsorption experiments were obtained by diluting the stock solution. HCl, NaOH, FeCl3, PbCl2, and oleic acid (supplied by Sigma–Aldrich) were analytical grade.

The batch adsorption experiments were performed in closed glass flasks of 100 mL filled with the solutions of pH-adjusted adsorbate at ambient temperature. The phenol losses by vaporizing was hindered by immediate sealing of flasks^34^. The pH of aqueous phase was changed with drops of HCl and NaOH (both are 0.1 M). All samples were filtered after equilibration to prevent interfering the carbon fines with the analysis. Finally, the concentrations of phenol in the supernatant solutions were determined by UV-spectrophotometry (271 nm). The results presented in the manuscript were based on the average of three tests, which were carried out three times for each condition.

To calculate the amount of the phenol adsorbed by sorbent (${\mathrm{q}}_{\mathrm{e}}$) the following expression was applied:

1 $${\mathrm{q}}_{\mathrm{e}}=\frac{({\mathrm{C}}_{0}-{\mathrm{C}}_{\mathrm{e}})\mathrm{V}}{\mathrm{m}}$$

in which, the adsorbate concentration at initial and equilibrium states are denoted by C_0_ and C_e_ in mg/L, V is the solution volume in L, and the grams of the sorbent materials is shown by m.

### MSAC preparation

A mixture of 25 mL of 0.4 M Fe^3+^ solution plus 25 mL of 0.2 M Pb^2+^ solution was prepared. 2.5 g AC was added into the Pb^2+^/Fe^3+^ solution during a stirring stage^37^. NaOH was then added dropwise until pH became greater than 12. Following this stage, the Oleic acid was added^42^. The resulted precipitate was centrifuged and was then washed repeatedly with ethanol and water to obtain the MSAC adsorbent. The process was followed by a drying stage in an oven at 60 °C. Then, the obtained composite was pyrolysized in a high temperature of 700 °C under argon atmosphere. The time and the process rate was 1 h and 10 °C/min.

The high resolution pictures of the developed sorbent was prepared by Scanning electron microscopy (SEM) (Seron Technology, AIS2100). The elemental composition of the developed adsorbent was detected by energy dispersive X-ray (EDX, Shimadzu). For SEM and EDX analysis, around 50 mg mass of adsorbent was applied. The surface chemical properties of developed adsorbent were detected by FTIR spectroscopy (Shimadzu IR instrument). The samples (MSAC before and after adsorption) were prepared in KBr discs in the usual way from alright dried mixtures of about 4% (w/w). FTIR spectra were recorded by the buildup of a minimum of 64 scans with a resolution of 4 cm^−1^ per sample. BET analysis was applied to sepcify the pore size and specific surface area of the developed MSAC using Belsorp mini II model. Around 50 mg mass of each adsorbent is applied for BET analysis. In this experiment, the sample tube was submerged into the liquid N_2_ reservoir at 77 K during the N_2_ gas adsorption. Thermo-gravimetric analysis (using PT1000, LINSEIS, Germany) was conducted under sample heating from 20 to 900 °C at the heating rate of 10 °C/min. In this experiment, around 5 mg mass of adsorbent was used. The experiment was conducted in an N_2_ atmosphere from 20 to 800 °C and using a synthetic air atmosphere from 800 to 900 °C.

## Adsorption modeling

### Adsorption equilibrium

To further explore the adsorption mechanism, the adsorption isotherms of phenol onto MSAC and pristine activated carbon adsorbents were investigated using Tempkin^43^, Freundlich^44^, and Langmuir^39^ isotherm models which their equations are as follows:

2 $${\text{Tempkin}}\;q_{e} = B\ln (K_{T} C_{e} );\;\;\;B = RT/b_{T}$$

3 $${\text{Freundlich}}\;q_{e} = K_{F} C_{e}^{1/n}$$

4 $${\text{Langmuir}}\;q_{e} = \frac{{q_{m} K_{L} C_{e} }}{{1 + K_{L} C_{e} }}$$

where *K*_*T*_ is constant with the unit of reciprocal of concentration (L/mg), and interconnected to maximum binding energy; *B* is the Tempkin constant corresponding to the adsorption heat (mg/g); *1/n* is known as the exponent of Freundlich; *K*_*F*_ is Freundlich model constant related to the adsorption capacity of adsorbent (mg/g)(mg/L)^−1/n^; and *K*_*L*_ and *q*_*m*_ are Langmuir constant (L/mg) and the theoretical saturation capacity (mg/g), respectively.

### Adsorption thermodynamics

Temperature significantly affects the adsorption process. Therefore, the adsorbent properties could be modified, and the phenol properties in solutions could change. The thermodynamic parameters include $\Delta$G (Gibbs free energy change of adsorption) (kJ/mol), $\Delta$S (entropy change of adsorption) (kJ/mol K), and $\Delta$H (enthalpy change of adsorption) (kJ/mol), which can be calculated using equilibrium constants at diverse temperatures. These parameters are obtained as follow^45^:

5 $$\Delta \mathrm{G}= \Delta \mathrm{H}-\mathrm{T}\Delta \mathrm{S}$$

6 $$\mathrm{K}=\frac{{\mathrm{C}}_{\mathrm{a}}}{{\mathrm{C}}_{\mathrm{b}}}$$

7 $$\mathrm{lnK }=-\frac{\Delta \mathrm{H}}{\mathrm{RT}}+\frac{\Delta \mathrm{S}}{\mathrm{R}}$$

where K is the thermodynamic equilibrium constant (–), T is the temperature (K), R is the universal gas constant (kJ/mol K), C_a_ and C_b_ (mg/L) are the equilibrium concentration of phenol on the adsorbent and adsorbate phases, respectively.

### Statistical physics evaluation

To theoretically understand the adsorption mechanism of phenol, statistical physics modeling was applied. This method assumes that a changeable amount of molecules is linked to N_M_ receptor sites of the adsorbent (unit mass)^31^. The reaction of adsorption for adsorbate molecules (P) onto receptor sites of adsorbent (M) is as follows:

8 $$\mathrm{nP}+\mathrm{M}\to {\mathrm{P}}_{\mathrm{n}}\mathrm{M}$$

where n is the stoichiometric coefficient of reaction, the n parameter can be an integer value or not. The values of n smaller than the unity indicate that the multi-anchorage adsorption mechanism may occur, while those greater than the unity represent the assumption of a multi-molecular adsorption mechanism^46^. In this context, a single layer adsorption process and or two or more adsorption layers may be considered adsorption isotherm. This work used two sta-phy models: single-layer (Eq. 6) and double-layer (Eq. 7). The variation of the adsorbed quantity of adsorbate (Q (mg/g)) against the equilibrium concentration (C (mg/L)) for these models is given by:

9 $$\mathrm{Q}=\frac{{\mathrm{nN}}_{\mathrm{M}}}{1+{\left(\frac{{\mathrm{C}}_{1/2}}{\mathrm{C}}\right)}^{\mathrm{n}}}$$

10 $$\mathrm{Q}={\mathrm{nN}}_{\mathrm{M}}\frac{{\left(\frac{\mathrm{C}}{{\mathrm{C}}_{1}}\right)}^{\mathrm{n}}+{2\left(\frac{\mathrm{C}}{{\mathrm{C}}_{2}}\right)}^{2\mathrm{n}}}{1+{\left(\frac{\mathrm{C}}{{\mathrm{C}}_{1}}\right)}^{\mathrm{n}}+{\left(\frac{\mathrm{C}}{{\mathrm{C}}_{2}}\right)}^{2\mathrm{n}}}$$

where n denotes the number of phenol molecules connected per receptor sites of the developed adsorbent; C_1/2_ is the concentration at half-saturation; N_M_ indicates the receptor areas density of adsorbent; Q_sat_ is the absorbed amount of molecule at saturation which is equal to:

11 $${\mathrm{Q}}_{\mathrm{sat}}={\mathrm{nN}}_{\mathrm{M}}\left(1+{\mathrm{N}}_{2}\right)$$

In the monolayer adsorption process, N_2_ = 0.

C_1_ and C_2_ are respectively the first and second layer concentrations at half-saturation.

## Results and discussion

### MSAC characterization

**Figure 1:**
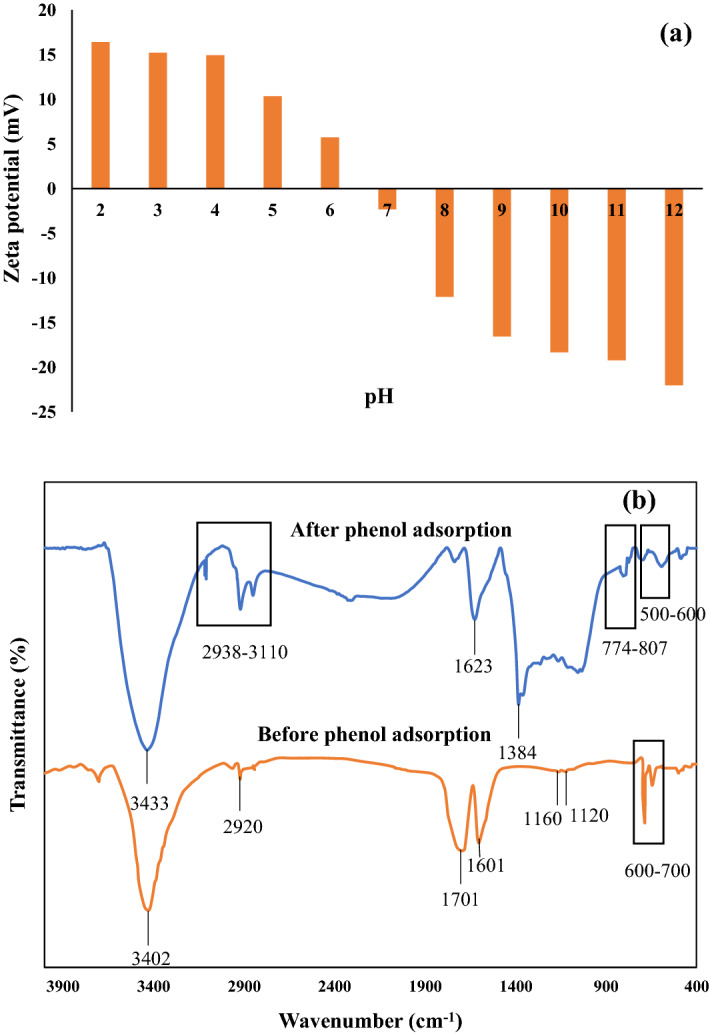
(**a**) Zeta potential and (**b**) FTIR spectra (transmitance mode) of MSAC composite before and after phenol adsorption (T = 25 °C).

Figure 1a shows the zeta potential on the surface of MSAC. The pH_pzc_ value of MSAC being 6.7 causes net charge of the developed adsorbent below this pH to be positive. At pH > pH_pzc_, the net charge of the developed adsorbent is negative. Therefore, protonated species will attract each other, and thus, the adsorption yield will increase.

FTIR spectra of adsorbent were conducted to detect the surface chemical properties of MSAC adsorbent before and after the adsorption of phenol (Fig. 1b). The observed peak at 1601 cm^−1^ was attributed to the bending vibration of H–O. In addition, the adsorbed water has usually shown a peak in the region of 3600–3200 cm^−1^ owing to stretching vibration of H–O, ${\text{v}}_{\text{s}}$ OH, as shown by 3402 cm^−1^ in the FTIR spectra. The peaks emerged at 2920 was related to methylene group asymmetric (${\text{v}}_{\text{as}}{{\text{CH}}}_{2}$), and methylene group symmetric (${\text{v}}_{\text{s}}{{\text{CH}}}_{2}$) stetching vibration. The small sharp bands revealed at 2940–2840 cm^−1^ region was resulted from C–H stertching vibration (${\text{v}}_{\text{s}}$ CH). For AC, the vibration of O–C–O and C–O usually produces bands in the 1180–1110 cm^−1^ region^47^. The presence of the lead and ferrite was confirmed by the observed bands at 700–600 cm^−1^ attributed to ${\text{v}}_{\text{s}}$ Fe-oxide and ${\text{v}}_{\text{s}}$ Pb–O-Pb. However, following the adsorption process these vibration peaks of metal–oxygen bonds were subjected to a small shift from 643 to 584 cm^−1^, and 687 to 588 cm^−1^, respectively. The phenol adsorption onto AC was demonstrated through the band apperaing at about 1384 cm^−1^. In addition, the emergence of new bonds at the region of 3110–2938 cm^−1^ can be assigned to the C–H peak of phenol. Also, the adsorbed phenol produced some additional bands in the fingerprint region (centered at 807 cm^−1^ and 774 cm^−1^) that proved a mono-substitution aromatic.

**Figure 2:**
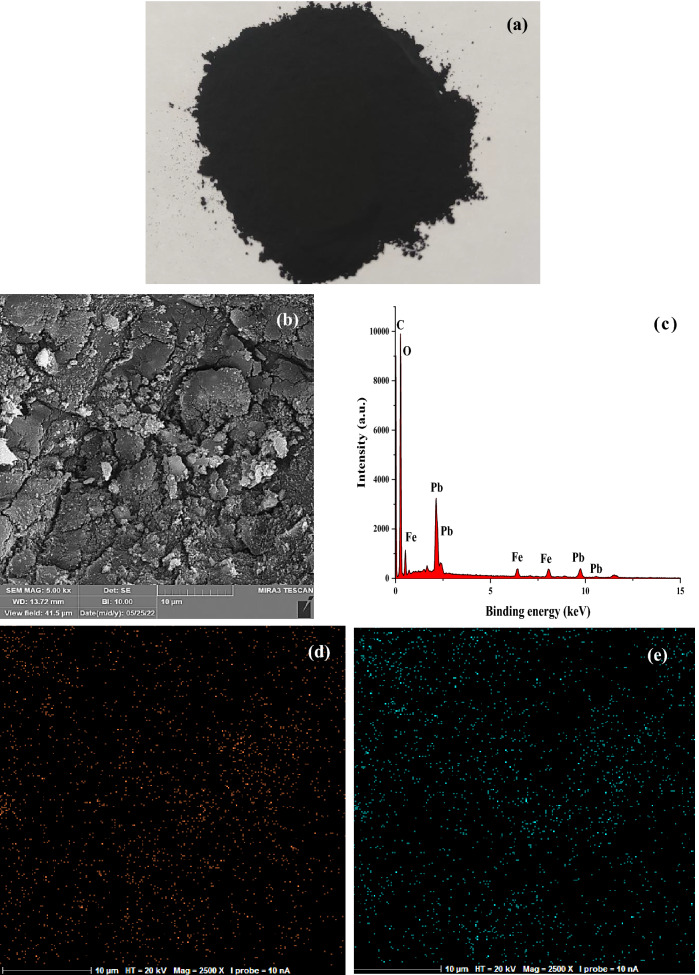
(**a**) Real picture, (**b**): SEM image, (**c**): EDX analysis of MSACadsorbent, and X-ray mapping of (**d**): lead, and (**e**): iron in MSAC.

The morphology of MSAC adsorbent (Fig. 2a) was investigated by scanning electron microscopy. The SEM photograph coupled with X-ray mapping for the developed adsorbent are presented in Fig. 2b–e. Figure 2b shows the aggregation of the precipitates of Pb and Fe on the AC surface. From Fig. 2d,e illustrate the individual detection of Pb and Fe elements on the AC surface. In fact, these elements were homogeneously distributed on the prepared absorbent surface. To further understand the individual component percentages in the MSAC composite, Scanning electron microscopy-energy-dispersive X-ray spectroscopy (SEM–EDX) analysis was performed (Fig. 2c). EDX spectrum of the MSAC composite shows that the percentages of Pb and Fe were 14.96% and 7.21%, respectively, that of carbon was 69.94% and that of oxygen was 7.79%. The determined atomic ratio of Pb to Fe in the developed adsorbent was 1:2.02, which was in close proximity to the theoretical ratio in PbFe_2_O_4_. The results of BET analysis showed the pristine AC and MSAC have specific surface area of 1023.9 m^2^/g and 774.53 m^2^/g, respectively. Also, the pore size values of pristine AC and MSAC composite were 4.013 nm and 11.89 nm, respectively. The change in specific surface area and pore size of MSAC composite could be attributed to the blockage of some pores following the precipitation of Pb and Fe on the surface of activated carbon. The blockage was more likely to be occurred for small pores. Therefore, as the number of small pores in the course of MSAC preparation decreased, the specific surface area was reduced. However, the remaining relatively larger pores resulted in higher value for pore size of MSAC, as compared to pristine AC.

Literature review indicated the phenol adsorption is governed mainly by micropore filling through the π–π dispersion interaction in micropores smaller than double molecular diameter of phenol^48^. Taking into account phenol molecular size (0.43–0.57 nm) and the pore size values of pristine AC and MSAC composite that are 4.013 nm and 11.89 nm, respectively, it can be found that the molecular size of phenol is less than the pore size obtaining for both sorbents. Therefore, the phenol molecules transference inside the adsorbent is ensured.

**Figure 3:**
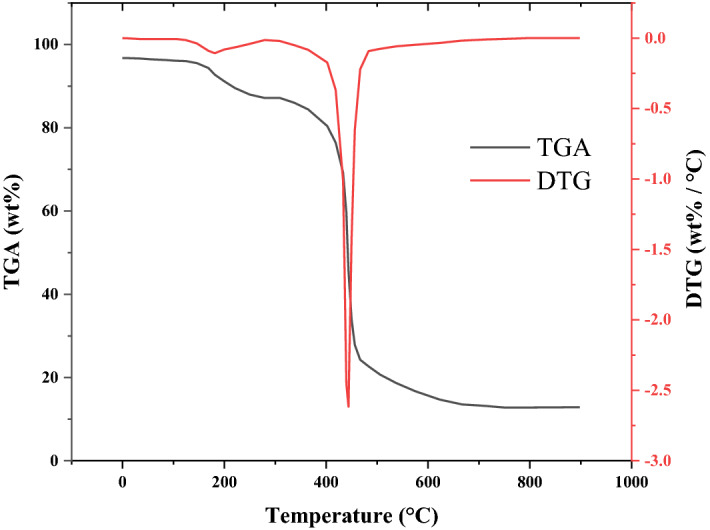
TGA and DTG curves for MSAC adsorbent.

TGA analysis was applied to study the thermo-stability of organic/inorganic components in the developed MSAC adsorbent against the heating in a range of 20 °C to 900 °C. Stepwise heating process was performed in a nitrogen atmosphere at a rate of 10 °C/min. In addition, the TGA curve achieved continuously during the mass loss was also differentiated, which provides the derivative thermo-gravimetric (DTG) plot (Fig. 3). A slight weight loss at the initial stage of heating (in temperature range less that about 100–150 °C) is usually observed in some cases and can be due to the burn-off of volatile constituents. A significant weight loss is occurred after the initial stage in the temperature range of 200–450 °C, indicated by a sharp peak in DTG curve. At the final stage, by completion of the heating the metal compounds are retained.

Analysis of thermo-gravimetric curves demonstrated three main portions of constituents for the developed MSAC: the higher portion composed of carbon-based material (70% by weight), the second one includes 9% weight volatile materials, and the third portion comprised of material with higher thermos-stability such as metals and other impurities.

**Figure 4:**
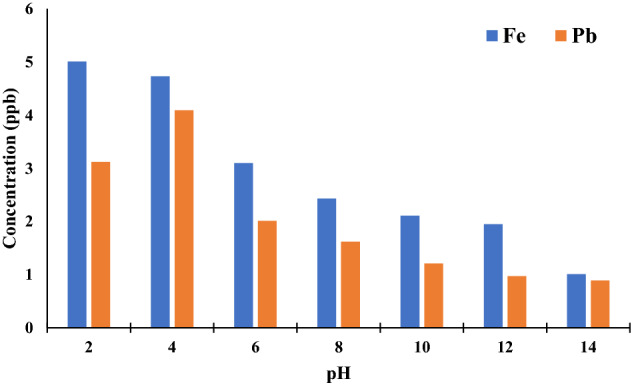
Concentration of lead and iron in the leach liquor solution at different pH values (pH = 2–14, T = 25 °C).

Environmental Protection Agency (EPA) guidelines state that Pb and Fe in drinking water should not exceed 50 and 100 μg/L, respectively. In this regard, the adsorbent and water were mixed together for twenty-four hours using a magnetic stirrer at various initial pH values, ranging from 2 to 14. As seen in Fig. 4, the concentrations of lead and iron at each of the four pH values were far lower than what is considered acceptable.Therefore, it should be possible to successfully use this highly stable adsorbent for the purposes of treating wastewater.

### Thermodynamic interpretation

**Figure 5:**
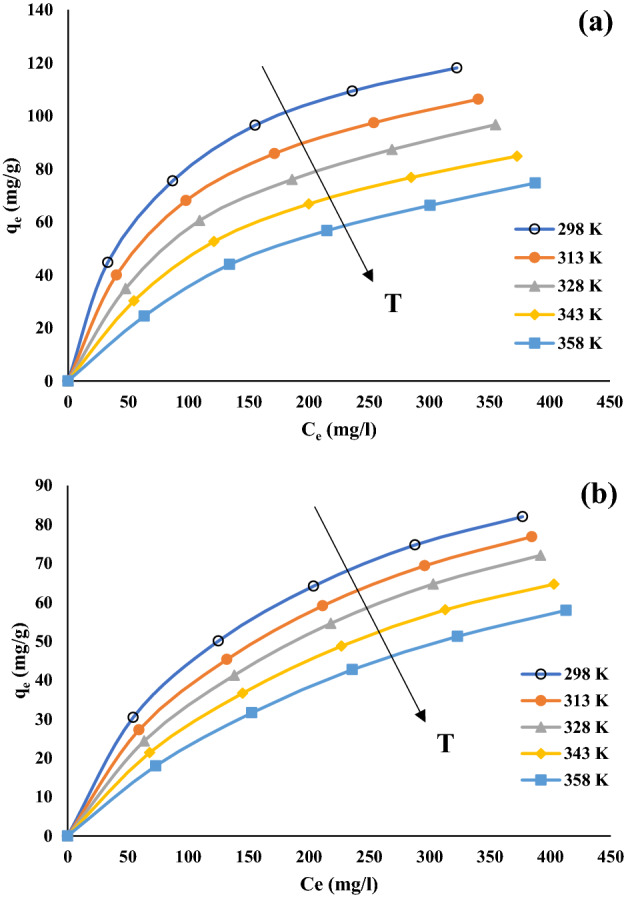
Influence of temperature on phenol adsorption isotherms onto (**a**) MSAC and (**b**) pristine activated carbon (Initial phenol concentration = 100 mg/L, pH = 7 and adsorbent dosage = 1.5 g/L).

Figure 5 shows the effect of temperature on the phenol adsorption onto MSAC composite and unmodified activated carbon at different initial phenol concentrations, i.e., the isotherm curves. The curves presented a favorable profile, with high phenol adsorption capacities even at low equilibrium concentrations. At C_e_ values lower than 200 mg/L, the curves are more inclined, revealing a good affinity between phenol and the magnetic activated carbon. It can be seen that, as C_e_ increases, this inclination decreases, and q_e_ tends to a constant value (the plateau). This profile is common for the temperature range from 298 to 358 K.

**Figure 6:**
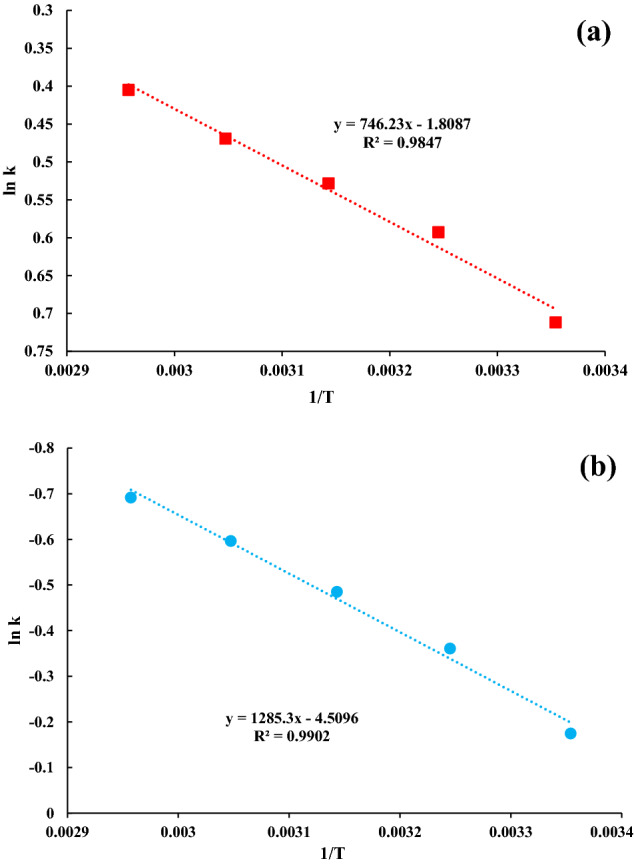
Vant’ Hoff plot for the phenol adsorption onto (**a**) MSAC and (**b**) pristine activated carbon.

As the temperature of the process increased, the amount of phenol adsorbed onto both adsorbents, MSAC and pristine AC, decreased. Figure 6 shows the plotting ln K versus [1/T] for 100 mg/L. Table 1 displays the thermodynamic parameters of the phenol adsorption onto MSAC and pristine activated carbon. An increasing trend of temperature from 298 to 358 K during the adsorption process revealed negative amounts of ΔH and ΔG, indicating the spontaneous and exothermic nature of the process. Also, the negative ∆S exhibited the stability of the solution–solid interface during the adsorption of phenol onto MSAC composite. The negative values of ΔG indicated the spontaneous adsorption of phenol onto the developed adsorbent. Therefore, it was deduced that phenol adsorption onto MSAC at low temperatures is favored (Fig. 6).

**Table 1:** Thermodynamic parameters of phenol adsorption onto MSAC and pristine activated carbon at different temperature.

Adsorbent	ΔH (kJ/mol)	ΔS (J/mol K)	ΔG (kJ/mol)				
			298 K	313 K	328 K	343 K	358 K
MSAC	− 6.20	− 15.04	− 1.72	− 1.57	− 1.42	− 1.27	− 1.12
Pristine AC	− 10.69	− 37.49	0.49	0.87	1.24	1.62	1.99

As the temperature of the process increased, the adsorption of phenol onto pristine activated carbon decreased. As given in Table 1, positive ΔG values describe that adsorption is non-spontaneous under examined conditions. In the case of pristien AC application for phenol adsorption, an increase in temperature from 298 to 358 led to the negative amounts of ΔH and ΔS, confirming the exothermic and associative nature of the process. Results of the thermodynamic studies for two adsorption processes of phenol onto MSAC and pristine activated carbon shows that lead ferrite coating on activated carbon causes the spontaneous adsorption process. In fact, lead ferrite coating on activated carbon changes the nature of the process from non-spontaneous toward spontaneous state.

Another thing is that the magnitude of ΔH was lower than 80 kJ/mol (Table 1). This observation is indicative that the phenol adsorption onto both adsorbents is controlled mainly by physical forces. Finally, it can be seen that the values of ΔG for MSAC composite increased with T, so the more negative value was found at 298 K, confirming that the phenol adsorption is favored at this temperature.

The possible explanation for this temperature-dependent behavior can be based on the adsorbent or adsorbate. It was encountered that the adsorbent is little affected by the temperature in this range (by analytical techniques and experiments). Its main characteristics, including surface chemistry and textural properties, keep the same from 298 to 358 K under aqueous media. In this sense, the temperature effect could be explained by the phenol characteristics. The temperature increase leads to an increase in phenol solubility. Consequently, phenol prefers to be water-diluted than be adsorbed into the adsorbent surface, leading to exothermic adsorption, as observed in this research.

### Equilibrium studies

**Figure 7:**
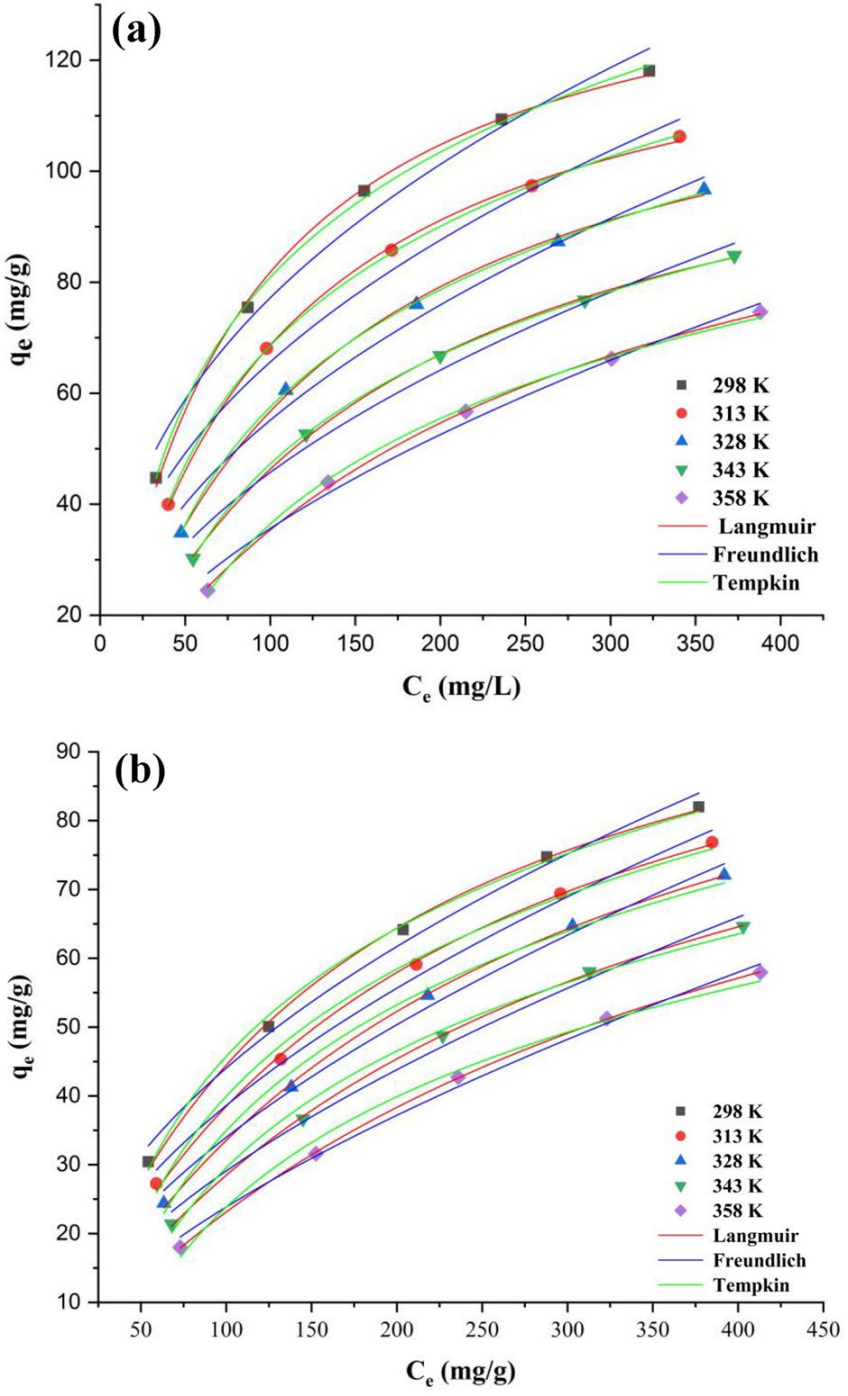
Adsorption isotherm curves for phenol removal using (**a**) MSAC and (**b**) pristine activated carbon (pH = 7, concentration = 1.5 g/L).

To better understand the detailed processes about the influence of temperature on the adsorption equilibrium, the experimental data were analyzed by Langmuir, Freundlich and Tempkin models. Figure 7 reveals the equilibrium curves for the adsorption of phenol onto MSAC composite and pristine activated carbon at different temperatures. It can be found that the adsorption capacity of both adsorbents decreased with an increase in temperature, being the highest capacities found at 298 K. Table 2 presents the isothermal parameters for the adsorption process in the case of both MSAC as well as pristine AC. The values of R^2^, R^2^_adj_ and MSE indicated that the Langmuir and Tempkin models can represent the experimental data for MSAC composite in all temperatures 298–358 K. Analyzing isothermal data indicated that the experimental data for pristine activated carbon were best fitted in the Langmuir, Freundlich and Tempkin models.

**Table 2:** Equilibrium adsorption parameters for phenol removal by MSAC and pristine AC.

Model	MSAC					Pristine AC				
	Temperature (K)					Temperature (K)				
	298	313	328	343	358	298	313	328	343	358
**Langmuir**										
*q*_*m*_ (mg/g)	145.71	135.62	131.09	120.94	120.31	116.604	117.274	118.547	111.827	111.973
*K*_*L*_ (L/mg)	0.0128	0.0103	0.0076	0.0062	0.0042	0.0062	0.0049	0.0039	0.0034	0.0026
*R*^*2*^	0.9986	0.9994	0.9986	0.9994	0.9989	0.9984	0.9989	0.9994	0.9997	0.9999
*R*^*2*^_*adj*_	0.9982	0.9992	0.9981	0.9992	0.9986	0.9979	0.9986	0.9992	0.9996	0.9999
*MSE* (mg/g)^2^	0.941	0.329	0.667	0.223	0.331	0.529	0.336	0.165	0.076	0.019
**Freundlich**										
*K*_*F*_ ((mg/g)(mg/L)^−1/n^)	12.713	9.627	6.587	4.811	2.693	4.688	3.395	2.495	1.919	1.249
*1/n* (dimensionless)	2.554	2.399	2.168	2.045	1.783	2.056	1.894	1.763	1.694	1.561
*R*^*2*^	0.9786	0.9796	0.9829	0.9827	0.9873	0.9911	0.9925	0.9929	0.9923	0.9938
*R*^*2*^_*adj*_	0.9715	0.9729	0.9773	0.9769	0.9831	0.9882	0.9901	0.9905	0.9897	0.9917
*MSE* (mg/g)^2^	14.778	11.283	8.054	6.445	3.945	2.995	2.331	2.065	1.851	1.262
**Tempkin**										
*K*_*T*_ (L/mg)	76.173	79.876	81.173	87.464	90.389	92.349	92.928	93.986	101.142	107.134
*b*_*T*_ (J/mol)	0.12	0.091	0.066	0.053	0.038	0.055	0.045	0.038	0.033	0.028
*R*^*2*^	0.999	0.9998	0.9997	0.9999	0.9985	0.9969	0.9951	0.9936	0.9935	0.9913
*R*^*2*^_*adj*_	0.9987	0.9998	0.9996	0.9999	0.9979	0.9959	0.9934	0.9915	0.9913	0.9884
*MSE* (mg/g)^2^	0.673	0.094	0.148	0.024	0.475	1.019	1.537	1.848	1.557	1.753

### Statistical physics inferences

Sta-phy models assessed new insights about the phenol adsorption onto both adsorbents. Single-layer (Eq. 6) and double-layer (Eq. 7) models were selected. The model selection was based on the coefficient of determination (R^2^) and the number of models parameters. Table 3 depicts the R^2^ values for both models in all tested temperatures. It can be found that both models agree with the experimental isotherms since good R^2^ values were found. However, a detailed analysis showed that the single-layer model presented higher R^2^ values for all temperatures. Besides, the double-layer model has four parameters, while the single-layer has only three.

**Table 3:** Coefficient of determination (R^2^) for different statistical physics models for MSAC and pristine activated carbon.

T (K)	Models for MSAC		Models for pristine AC	
	Single-layer	Double-layer	Single-layer	Double-layer
298	0.9999	0.9771	0.9999	0.9745
313	0.9999	0.9793	0.9999	0.9684
328	0.9999	0.9821	0.9999	0.9635
343	0.9999	0.9837	0.9999	0.9588
358	0.9999	0.9814	0.9999	0.9480

In conclusion, the single-layer model was considered as the best sta-phy model to predict the phenol adsorption isotherms on both adsorbents. Therefore, the sta-phy parameters estimated by this model were applied for adsorption systems interpretation based on this result. Thus, the parameters are n (number of phenol molecules per site), N_M_ (density of receptor sites), Q_sat_ (adsorbed amount at saturation), and E (adsorption energy). Table 4 lists the mentioned parameters.

**Table 4:** Statistical physics parameters for the phenol adsorption on MSAC and pristine activated carbon based on the single-layer model.

T (K)	MSAC				Pristine AC			
	N	N_M_ (mg/g)	Q_sat_ (mg/g)	E (kJ/mol)	n	N_M_ (mg/g)	Q_sat_ (mg/g)	E (kJ/mol)
298	0.88	180.72	158.94	16.75	0.85	163.43	138.91	14.45
313	0.93	152.97	142.61	17.25	0.86	158.37	138.54	14.65
328	0.96	142.53	136.22	17.35	0.92	145.19	133.36	15.10
343	1.02	116.65	118.91	17.91	0.97	121.55	117.45	15.81
358	1.04	111.02	115.23	17.66	0.99	112.56	112.34	15.98

#### Statistical physics model parameters (n and N_M_)

**Figure 8:**
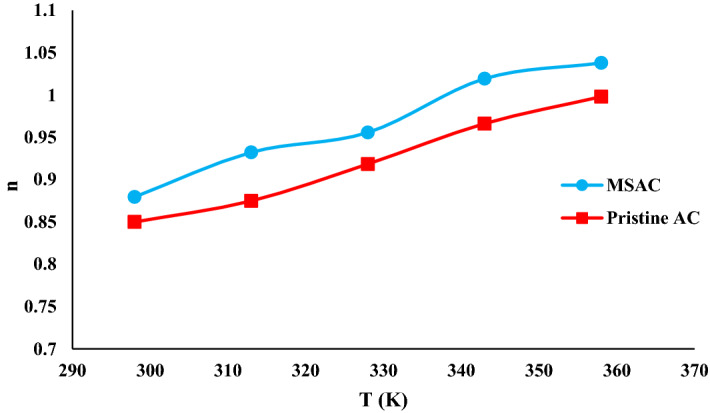
Change in n parameter with the variation of temperature for MSAC and pristine AC.

The examination of the number of phenol molecules linked per adsorbent receptor site provides valuable data to understand the adsorption mechanism of phenol on the adsorbent. This parameter can outline the geometrical position of phenol at different temperatures. It is enough to compare the values of n by the unity to identify the adsorption position. If the values of n are smaller than one, it describes the position of adsorption as a parallel because a part of phenol molecules is linked to the adsorbent. On the other hand, if the values of n are equal or higher than one, one or more phenol molecules are linked to each binding site of the adsorbent^31^. The effect of temperature on the number of phenol molecules linked per adsorbent site is shown in Fig. 8.

The values of n changed from 0.88 to 1.04 for the adsorption system of phenol- MSAC (Table 4 and Fig. 8). The value of n greater than one implied that the active site of MSAC adsorbent interacted with more than one phenol, and therefore inclined orientation was deduced^31^. The main adsorption site of the MSAC could capture a fraction of phenol molecule when n < 1. The later suggested that the orientation of molecules attached to the adsorbent surface was horizontal^31^. The behavior of this parameter showed that the phenol orientation on MSAC composite was changed as n raised up to 1.04 (Fig. 8). This phenomenon could be assigned to thermal agitation. Moreover, the physicochemical properties of the developed adsorbent is supposed to be responsible for the transition of phenol adsorption. In fact, the molecules of phenol establish a multi-molecular interaction with the surface of adsorbent. Also, the transition of phenol molecule on the adsorbent surface from parallel to vertical position can be possibly related to the adsorption of –OH group on the functional groups of the developed adsorbent.

**Figure 9:**
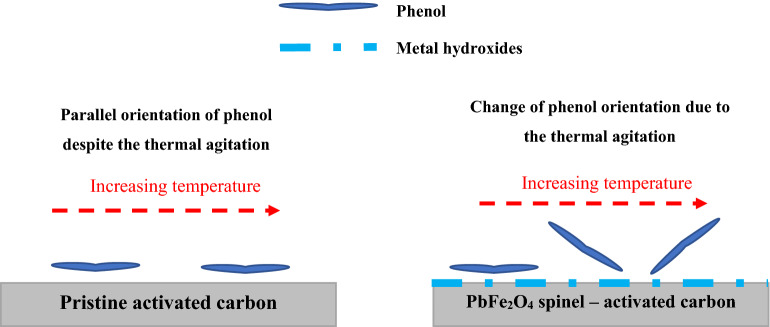
Schematic diagram for the change of phenol orientation onto MSAC and pristine activated carbon due to thermal agitation.

As given in Table 4, the values of n for the adsorption system of phenol-pristine AC varied from 0.85 to 0.99. Considering the values of n for the mentioned system (n < 1), it can be found that pristine AC could capture a fraction of phenol molecule. Also, it was concluded that phenol molecule was docked with parallel orientation on pristine AC. The behavior of this parameter indicated that phenol molecule could not move freely on the surface of tested adsorbent (Fig. 9).

**Figure 10:**
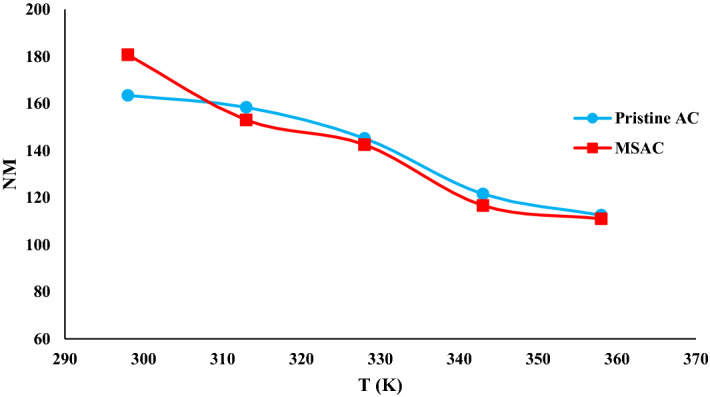
Dependence of the density of receptor sites on temperature for MSAC and pristine activated carbon.

The density of receptor sites (N_M_) of tested adsorbent is an important parameter for the uptake of phenol onto the adsorbent. Figure 10 shows the evolution of the density of receptor sites of MSAC composite and pristine activated carbon at various temperatures. It can be found that the number of anchorages increased upon raising the temperature. This behavior expresses an antagonistic effect. As the amount of phenol molecules per site enhances, the accessible active site of the adsorbent for phenol removal decreases.

#### Adsorbed amount at saturation (Q_sat_)

**Figure 11:**
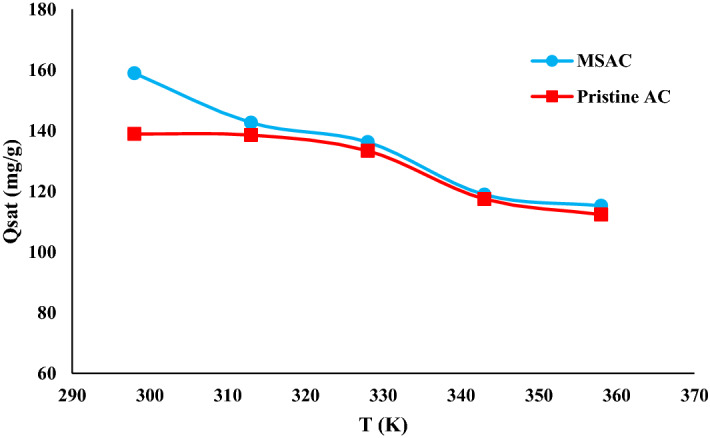
Influence of temperature on adsorbed quantity at saturation for MSAC and pristine activated carbon.

The parameter of adsorbed quantity at saturation provides valuable information about the adsorbent, which can be affected by external variables (pH, adsorbate concentration, and temperature). Figure 11 shows the change in the amount of adoption at the saturation state as temperature varies. From an analytical point of view, its values are equal to the product of the two parameters, n, and N_M_. As given in Table 4, as the temperature rose from 298 to 358 K, the value of Q_sat_ for MSAC and pristine AC decreased from 158.94 and 138.91 to 115.23 and 112.34 mg/g indicating that the phenol adsorption onto both mentioned adsorbents is exothermic.

Results indicated that at all examined temperatures, the adsorption quantities at saturation for pristine activated carbon were smaller than those of MSAC composite, confirmed the experimental data. This discrepancy is related to a variation in the structure of MSAC as it has additional metallic hydroxides in comparison with pristine AC. These hydroxides can facilitate the adsorption of phenol through establishment of a stronger interaction.

#### Adsorption energy (E)

It is essential to calculate the adsorption energy during the adsorption process to identify the phenol removal mechanism. The C_1/2_ (i.e., half-saturation concentration) is associated with the binding energy of adsorbed layer, considering the expression of the monolayer adsorption process. From this parameter (C_1/2_), the adsorption energy value can be estimated and used to interpret the temperature effect. The expression of adsorption energy is written as:

12 $$\mathrm{E}=\mathrm{RTln}\left(\frac{{\mathrm{C}}_{\mathrm{s}}}{{\mathrm{C}}_{1/2}}\right)$$

where C_s_ denotes the solubility of phenol in water.

**Figure 12:**
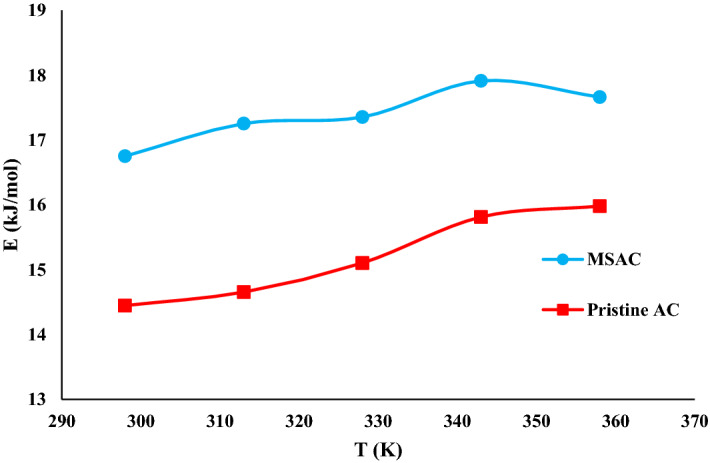
Influence of temperature on adsorption energy (E) for MSAC and pristine activated carbon.

Table 4 presents the adsorption energies of phenol onto both adsorbents, which were relatively smaller than 20 kJ/mol. Figure 12 shows the evolution of adsorption energy at various temperatures. The obtained findings revealed that the adsorption of phenol from the aqueous phase using MSAC composite and pristine AC could occur via physical adsorption. Furthermore, as the temperature of the process increased, the adsorption energy increased, indicating that the nature of phenol adsorption was exothermic, which was agreement with the experimental data.

According to the interpretation of single-layer model, it was concluded that the principal reason that enhanced the adsorption of phenol onto MSAC composite compared with the pristine AC is the metal hydroxides coated on activated carbon. These metal hydroxides from MSAC composite can contribute to a strong interaction with phenol molecule compared to the interactions between pristine AC and phenol molecules. In fact, the dominant mechanism for the adsorption of phenol on MSAC is attributed to the attractive electrostatic interaction that emerges between the metal hydroxides and phenol’s –OH acidic group. It is noted that all the adsorption capacities of phenol onto MSAC are higher than those of pristine AC due to the stronger interactions between phenol molecule and MSAC composite, especially the additional interaction provided by metal hydroxides.

### Proposed mechanism for the interaction of phenol with MSAC

**Figure 13:**
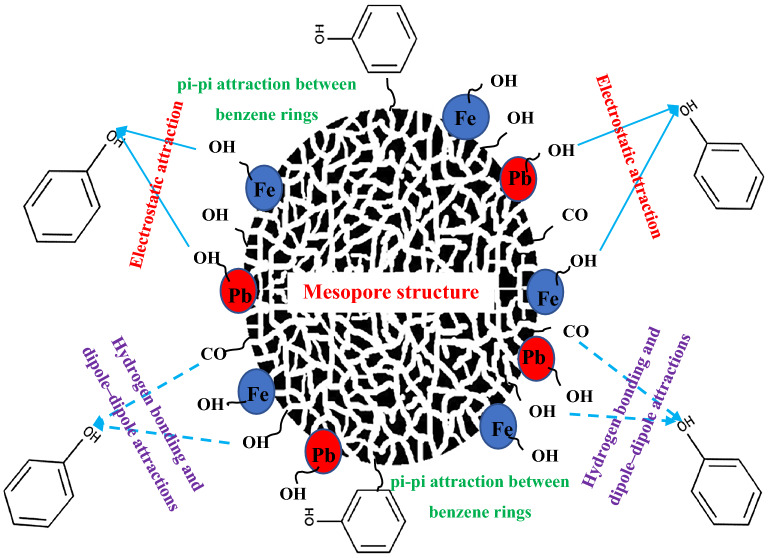
Proposed mechanism for phenol adsorption onto MSAC composite.

There are various proposed mechanisms such as electrostatic attraction, pi-pi attraction between the phenolic ring and activated carbon basal planes, donor–acceptor complex formation, and hydrogen bonding between phenol molecules, and the presence of suitable functional groups on the adsorbent surface^21,49^. The attractive electrostatic interaction of metal hydroxides and phenol’s –OH may affect the adsorption of phenol onto MSAC composite. Mechanistic studies showed that the values of the number of phenol molecules linked per adsorbent receptor site varied from 0.88 to 1.04 for the adsorption system of phenol- MSAC composite. In fact, the transition of phenol adsorption position was interconnected to the physicochemical characteristics of the developed adsorbent. The molecules of phenol establish a multi-molecular interaction with the surface of adsorbent. Also, the transition of phenol molecule on the adsorbent surface from parallel to vertical position can possibly be related to the adsorption of –OH interacting with the functional groups of the developed adsorbent. The latter is responsible for the higher adsorption quantities at saturation for MSAC composite than those of pristine activated carbon. This discrepancy is related to the structure of the adsorbent, as MSAC composite has additional –OH groups comparing to pristine AC. These groups enhance the phenol removal by creating a strong interaction with phenol molecules. In addition, functional groups on the AC surface may participate in the adsorption process. H-bond formation between these functional groups (i.e., OH and CO) (H-bond acceptor) phenol’s –OH (H-bond donor), and also dipole–dipole attractions are the dominant interactions of phenol with the adsorbent. In addition to the electrostatic attraction, the interaction of phenol atomic rings with the adsorbent surface through pi-pi electron donor–acceptor interactions can result in the phenol adsorption by the developed adsorbent. The proposed mechanism for phenol adsorption onto MSAC composite is shown in Fig. 13.

### Reusability of PbFe_2_O_4_ spinel-activated carbon composite

**Figure 14:**
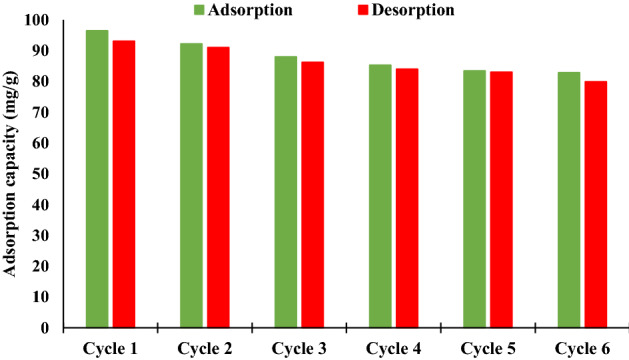
Adsorption capacity of phenol onto MSAC composite in successive cycles (C_0_ = 300 mg/L, pH = 7, concentration = 1.5 g/L).

Reusability of an adsorbent is an important parameter in the adsorption process. There are different methods, such as heating or chemical regeneration and solvent washing for reusing the adsorbent. In this research, solvent washing, as a well-known technique, was used to regenerate the PbFe_2_O_4_ spinel-activated carbon composite. The regeneration of developed adsorbent for six successive cycles (adsorption–desorption) was carried out by using 1 mol/L NaOH. Figure 14 shows the phenol removal capacity of MSAC composite during different adsorption/desorption cycles. Results indicted that the sorbent preserved 85 percent of its maximum capacity after these six cycles. The interesting adsorption potential of MSAC composite after six cycles of regeneration makes it practically/commercially attractive and green.

## Conclusion

This research reports the theoretical analysis of phenol adsorption onto PbFe_2_O_4_ spinel—magnetic activated carbon (MSAC) and pristine activated carbon. The main characteristics of MSAC are a surface area of 774.53 m^2^/g, a pore size of 11.89 nm, particle diameter lower than 0.25 mm, and a zeta potential of 6.7. The main functional groups on the surface are OH, aromatic rings, COC, CH_2_, Pb–O–Pb, and Fe–O–Fe. The adsorption efficiency of MSAC composite decreased with increasing temperature, indicating the exothermic and associative nature of the process. Comparison of the thermodynamic parameters of two adsorption process of phenol onto MSAC and pristine activated carbon shows that lead ferrite coating on activated carbon change the nature of the process from a non-spontaneous toward a spontaneous state. Furthermore, to theoretically understand the adsorption mechanism of phenol, statistical physics modeling was applied. The results indicated that a good relationship resulted from the single-layer model. After that, n, N_M_, Q_sat_, and E parameters were obtained from this model. The values of the number of phenol molecules linked per adsorbent receptor site varied from 0.88 to 1.04 for the adsorption system of phenol- MSAC. The behavior of this parameter showed a change of phenol orientation on the MSAC composite due to thermal agitation. Also, the values of n for the adsorption system of phenol-pristine AC varied from 0.85 to 0.99, indicating that pristine AC could capture a fraction of phenol molecules. It was concluded that the phenol molecule was docked with parallel orientation on pristine AC. By increasing the number of phenol molecules per site, the accessible active site of the developed adsorbent for phenol removal decreases. Therefore, variation of temperature from 298 to 358 K, the value of Q_sat_ decreased from 158.94 and 138.91 to 115.23 and 112.34 mg/g for MSAC and pristine AC, respectively. Also, the values of phenol adsorption energy were reduced, indicating that the phenol adsorption onto both mentioned adsorbents is exothermic. According to the interpretation of single-layer model, it was concluded that the principal reason that enhanced the adsorption of phenol onto MSAC composite compared with the pristine AC could be attributed to the functional role –OH groups coated on AC. These metal hydroxides from MSAC composite can contribute to establish a stronger interaction with phenols compared to the interactions between pristine AC and phenol molecules. The significant role of metallic hydroxides in MSAC to facilitate the removal of phenol is also confirmed by mechanistic studies. The main mechanism is the attractive electrostatic interaction of metal hydroxides with phenol’s –OH acidic group.

## Data Availability

The datasets used and/or analyzed during the current study available from the corresponding author on reasonable request.
